# Editorial: Application of psychological theories to the study of consumer and organizational behavior in a post-pandemic world

**DOI:** 10.3389/fpsyg.2023.1265474

**Published:** 2023-08-08

**Authors:** Yue Pan, Kathrin J. Hanek, Lujun Su

**Affiliations:** ^1^Department of Management & Marketing, School of Business Administration, University of Dayton, Dayton, OH, United States; ^2^Department of Marketing, Business School, Central South University, Changsha, Hunan, China

**Keywords:** consumer behavior, organizational behavior, pandemic, psychological theories, interdisciplinary research

The COVID-19 pandemic undoubtedly had far-reaching global repercussions on our everyday lives. This Research Topic showcases some of the psychological impacts, from person perception to consumer behavior and work lives that have been affected by social changes ushered in by the pandemic. Better understanding these shifts helps us not only examine the impacts of a global pandemic on psychological phenomena but also anticipate where consumer and organizational behavior may progress in a post-pandemic world. This Research Topic presents new insights and research advances in consumer and organizational behavior, and offers practical solutions for business executives to enhance consumption and organizational performance in the post-pandemic era. The Research Topic features six articles that investigate consumer and organizational behavior from different angles. Authors contributing to this Research Topic have applied a myriad of psychological theories to their studies of consumers' and employees' wellbeing, perceptions, attitudes, and behaviors.

Considering the impacts of the pandemic on our work lives and employees' occupational health, research by Weber et al. examines the factors that contribute to work fatigue during the lockdowns when people were teleworking. Their research highlights the multi-level influences—including gender, job demands, and work privacy fit—on how fatigued workers felt. By better understanding how environmental factors can contribute to work fatigue, their research also helps delineate ways to make post-pandemic work environments—especially the new normal of the home office—more conducive to employees' wellbeing.

The pandemic has disrupted supply chains worldwide and left some groups and suppliers particularly disadvantaged. As countries aim to alleviate issues of poverty generally and in the wake of the pandemic specifically, He's research focuses on China's “helping farmers” projects, which aim to support local farmers, to show how supply chain transparency of participating in such initiatives can affect consumers' perceptions of information quality and ultimately trust in the company. Thus, this research informs how post-pandemic consumers may be geared toward and psychologically respond to specific corporate social responsibility efforts.

The external stimuli triggered by the pandemic may affect the internal state of a consumer, eventually resulting in changes in their consumption behavior, in a way that may not be easily predicted a priori. Building on the stimulus-organism-response and health belief models, Leyva-Hernández et al. find that perceived severity (of the disease), perceived benefits, and cue to action positively and significantly affect social identity, and in turn, influence socially responsible consumption, while perceived barriers have a direct effect on socially responsible consumption. Different generation groups seem to display diverse patterns of sustainable consumption.

Research by Jeong et al. delves into a psycho-physiological research area somewhat unique to the world during a pandemic, attesting to something we may have known only anecdotally but not scientifically proved. Using eye-tracking devices, the authors provide empirical evidence for the urban myth of mask fishing effect. In particular, they've confirmed a curvilinear relationship between the amount of facial area covered by a face mask and the perceived attractiveness of an individual.

The pandemic has raised risk awareness among consumers, who are constantly exposed to risk information through various channels of information technology. Xue et al. examine how consumers cope with such risks and adopt self-protective behaviors in the midst of a pandemic. They propose a model that links the amount and credibility of risk information, consumers' self-protective willingness, risk perception, protective behavior attributes, and consumers' self-protective behavior. Their study contributes to the understanding of the role of risk information in influencing consumers' self-protective willingness, and extends the application of the Protective Action Decision Model.

Tourism is one of the sectors that have been most severely hit by the COVID-19 outbreak, with hotels being perhaps the most vulnerable subsector. SPR et al. analyze the factors that influence tourists' intentions to revisit hotels in the post-pandemic era, and find that service quality, promotional offers and perceived health risks play a significant role in shaping tourists' decisions. This study sheds light on how tourists evaluate hotels in a pandemic, reveals the underlying mechanisms of consumer repurchase behavior, and offers valuable insights for the hospitality industry to effectively increase the occupancy rate of hotels.

The articles presented in this Research Topic jointly serve as a springboard for interdisciplinary research that connects psychological theories with business problems. This Research Topic will induce further advancement of impactful and substantive research on consumer and employee coping strategies as they attempt to adapt to economic and social disruptions during a pandemic, as well as delve into the wide implications of environmental turbulence for consumers and organizations. Thus, the articles in this Research Topic contribute to the literature by shedding new light on some fundamental, yet challenging, questions that advance knowledge for both research and practice.

## Author contributions

YP: Conceptualization, Writing—original draft, Writing—review and editing. KH: Conceptualization, Writing—original draft, Writing—review and editing. LS: Conceptualization, Writing—original draft, Writing—review and editing.

